# Detection and Genetic Characterization of Community-Based SARS-CoV-2 Infections — New York City, March 2020

**DOI:** 10.15585/mmwr.mm6928a5

**Published:** 2020-07-17

**Authors:** Dena Bushman, Karen A. Alroy, Sharon K. Greene, Page Keating, Amanda Wahnich, Don Weiss, Preeti Pathela, Christy Harrison, Jennifer Rakeman, Gayle Langley, Suxiang Tong, Ying Tao, Anna Uehara, Krista Queen, Clinton R. Paden, Wendy Szymczak, Erika P. Orner, Priya Nori, Phi A. Lai, Jessica L. Jacobson, Harjot K. Singh, David P. Calfee, Lars F. Westblade, Ljiljana V. Vasovic, Jacob H. Rand, Dakai Liu, Vishnu Singh, Janice Burns, Nishant Prasad, CDC COVID-19 Surge Laboratory Group;, Jessica Sell

**Affiliations:** ^1^Incident Command System Surveillance and Epidemiology Section, New York City Department of Health and Mental Hygiene; ^2^Epidemic Intelligence Service, CDC; ^3^Public Health Laboratory, New York City Department of Health and Mental Hygiene; ^4^Division of Viral Diseases, National Center for Immunization and Respiratory Diseases, CDC; ^5^Department of Pathology, Montefiore Medical Center, Albert Einstein College of Medicine, Bronx, New York; ^6^Division of Infectious Diseases, Department of Medicine, Montefiore Medical Center, Albert Einstein College of Medicine, Bronx, New York; ^7^Department of Pathology and Laboratory Medicine, NYU Langone Hospital—Brooklyn, New York; ^8^New York City Health + Hospitals/Bellevue, New York, New York; ^9^Division of Infectious Diseases, Department of Medicine, Weill Cornell Medicine, New York, New York; ^10^Department of Pathology and Laboratory Medicine, Weill Cornell Medicine, New York, New York; ^11^New York-Presbyterian Queens, Flushing, New York.

To limit introduction of SARS-CoV-2, the virus that causes coronavirus disease 2019 (COVID-19), the United States restricted travel from China on February 2, 2020, and from Europe on March 13. To determine whether local transmission of SARS-CoV-2 could be detected, the New York City (NYC) Department of Health and Mental Hygiene (DOHMH) conducted deidentified sentinel surveillance at six NYC hospital emergency departments (EDs) during March 1–20. On March 8, while testing availability for SARS-CoV-2 was still limited, DOHMH announced sustained community transmission of SARS-CoV-2 (*1*). At this time, twenty-six NYC residents had confirmed COVID-19, and ED visits for influenza-like illness* increased, despite decreased influenza virus circulation.^†^ The following week, on March 15, when only seven of the 56 (13%) patients with known exposure histories had exposure outside of NYC, the level of community SARS-CoV-2 transmission status was elevated from sustained community transmission to widespread community transmission (*2*). Through sentinel surveillance during March 1–20, DOHMH collected 544 specimens from patients with influenza-like symptoms (ILS)^§^ who had negative test results for influenza and, in some instances, other respiratory pathogens.^¶^ All 544 specimens were tested for SARS-CoV-2 at CDC; 36 (6.6%) tested positive. Using genetic sequencing, CDC determined that the sequences of most SARS-CoV-2–positive specimens resembled those circulating in Europe, suggesting probable introductions of SARS-CoV-2 from Europe, from other U.S. locations, and local introductions from within New York. These findings demonstrate that partnering with health care facilities and developing the systems needed for rapid implementation of sentinel surveillance, coupled with capacity for genetic sequencing before an outbreak, can help inform timely containment and mitigation strategies.

The DOHMH collected deidentified remnant nasopharyngeal swab specimens from patients with ILS and no known virologic diagnosis evaluated at six sentinel EDs during March 1–20, 2020. Because of concern that SARS-CoV-2 could be introduced by travelers returning from China, where the outbreak originated, five EDs were selected because of their high use by patients residing in ZIP codes with ≥20% self-identified Chinese speakers.** Two EDs were in Manhattan, two in Queens, one in Brooklyn, and one in the Bronx. Refrigerated specimens were released to DOHMH 48 hours after collection, and frozen specimens were released 1 week after collection. Specimens collected during March 1–9 were from patients of all ages. Because little was known about pediatric SARS-CoV-2 infection, during March 10–20, DOHMH only collected specimens from patients aged <18 years.

Specimens were sent to CDC on March 23, 2020, for SARS-CoV-2 testing using the 2019-nCoV real-time reverse-transcription–polymerase chain reaction (RT-PCR) assay.^††.^ To conserve resources, pools with up to five specimens were tested together, and individual specimens within positive or inconclusive pools were retested. Nucleic acid from RT-PCR–positive specimens was then extracted and subjected to Oxford Nanopore MinION sequencing, and full genome sequences were generated using methods described previously (*3*). Phylogenetic relations were inferred using the Nextstrain pipeline (*4*), including the 36 positive SARS-CoV-2 sentinel specimens and selected full genome sequences available as of April 1, 2020, from the Global Initiative on Sharing All Influenza Data (GISAID) (*5*). This project was determined by DOHMH and CDC to be nonresearch public health surveillance. Therefore, approval by the agencies’ institutional review boards was not required.

Given limited testing availability, and to better understand prevalence of SARS-CoV-2 infections in the absence of NYC population prevalence data, DOHMH calculated the estimated weekly number of persons with undetected SARS-CoV-2 infection in the target population. DOHMH estimated^§§^ the weekly target population, defined as those persons evaluated at any NYC ED with ILS who had negative test results for influenza (and, in some instances, for other respiratory pathogens). Numbers of ED visits for ILS were obtained using ED syndromic surveillance data and aggregated weekly citywide and by sentinel ED. Each sentinel ED provided DOHMH their weekly influenza testing volume and results. Estimated SARS-CoV-2 prevalence among the target population was calculated using the estimated true prevalence tool^¶¶^ assuming 85% test sensitivity (range = 75%–95%) and 99% specificity of the SARS-CoV-2 RT-PCR assay; results were analyzed using R statistical software (version 3.6.3; The R Foundation).

During March 1–20, 544 specimens were collected from the six sentinel EDs (Table). Thirty-six (6.6%) specimens were positive for SARS-CoV-2, including 22 (5.2%) among 425 patients of all ages and 14 (11.8%) among 119 patients aged <18 years. Among the 36 SARS-CoV-2–positive specimens, 32 (89%) were obtained during two 3-day periods: March 8–10 and March 17–19 (Figure).

**TABLE:** Weekly emergency department (ED) sentinel surveillance results and SARS-CoV-2 prevalence estimations among persons with influenza-like symptoms (ILS) of all ages and those <18 years of age — New York City (NYC), March 2020

Characteristic	Age group			
	All ages		<18 yrs	
	Wk beginning Mar 1	Wk beginning Mar 8	Wk beginning Mar 8	Wk beginning Mar 15
ED visits for ILS citywide,* no.	17,137	24,511	7,546	4,464
ED visits for ILS at sentinel sites, no.	1,145	3,019	479	778
ED visits for ILS at sentinel sites with influenza tests performed, no. (%)^†^	449 (39.2)	1,606 (53.2)	440 (91.9)	252 (32.4)
ED visits for ILS at sentinel sites with negative influenza test results, no. (%)	336 (74.8)	1,275 (79.4)	328 (74.5)	224 (88.9)
Target population, no. of persons^§^	5,029	10,352	5,167	1,285
Sentinel surveillance specimens collected, no.	244	181	37	82
Specimens positive for SARS-CoV-2, no. (%)	3 (1.2)	19 (10.5)	1 (2.7)	13 (15.9)
Estimated SARS-CoV-2 prevalence in target population,^¶^ % (CL)**	0.3 (0.0–3.5)	11.3 (6.2–20.0)	2.0 (0.0–17.3)	17.7 (9.1–32.8)
Estimated undetected COVID-19 cases in target population, no. (CL)^††^	15 (0–176)	1,170 (642–2,070)	103 (0–894)	227 (117–422)
Confirmed COVID-19 cases in NYC,^§§^ no.	26	1,917	42	457

**Abbreviations:** CL = confidence limit; COVID-19 = coronavirus disease 2019; Wk = week.

* ILS are defined as having at least one of the following signs or symptoms recorded in the ED chief complaint: chills, fever, upper respiratory infection, cough, sore throat, runny nose, congestion, headache, or fatigue.

^†^ Limited to sentinel EDs that contributed samples during the specified week.

^§^ Target population is defined as those persons evaluated at any NYC ED with ILS who had negative test results for influenza (and, in some instances, for other respiratory pathogens). The target population is calculated using the following formula: ED visits for ILS citywide x (ED visits for ILS at sentinel sites with influenza tests performed/ED visits for ILS at sentinel sites) x (ED visits for ILS at sentinel sites with negative influenza test results/ED visits for ILS at sentinel sites with influenza tests performed).

^¶^ Point estimate calculated using estimated true prevalence tool (https://epitools.ausvet.com.au/trueprevalence), assuming 85% sensitivity and 99% specificity for the SARS-CoV-2 reverse transcription–polymerase chain reaction (RT-PCR) test for nasopharyngeal samples collected from symptomatic patients.

** Lower confidence limit calculated assuming 95% test sensitivity. Upper confidence limit calculated assuming 75% test sensitivity. All calculations assume 99% test specificity.

^††^ Calculated by multiplying target population by estimated prevalence and by lower and upper confidence limits.

^§§^ Confirmed cases are defined as having first positive SARS-CoV-2 RT-PCR test result reported to the NYC Department of Health and Mental Hygiene among NYC residents, as of June 18, 2020, with the specimen collected during the week specified.

**FIGURE:**
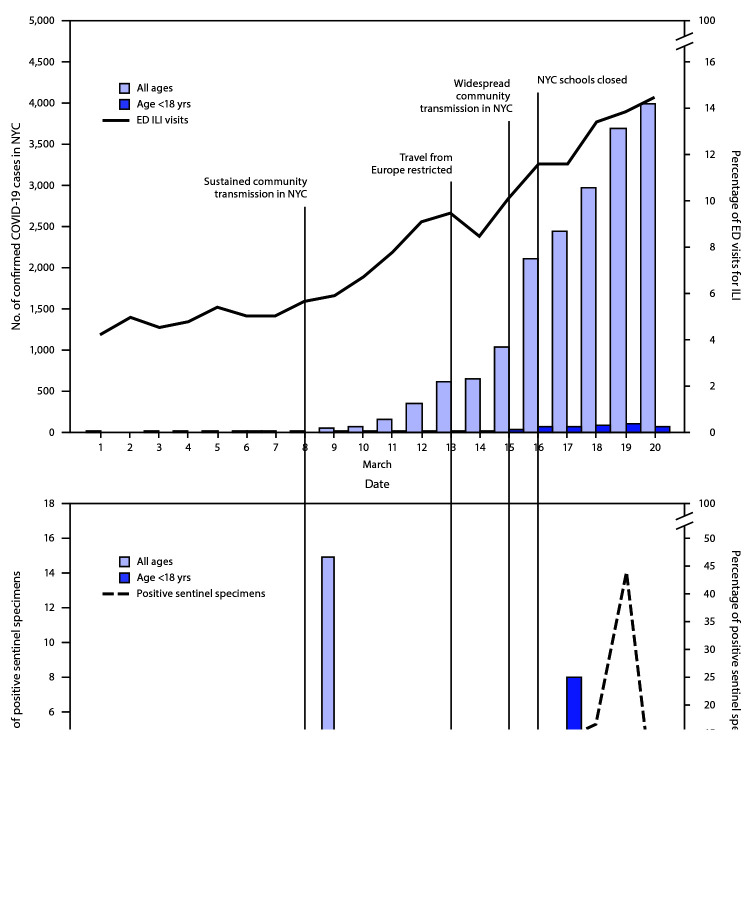
Daily percentage of emergency department (ED) visits for influenza-like illness (ILI), number of confirmed COVID-19 cases, and number and percentage of sentinel specimens positive for SARS-CoV-2* — New York City, March 1–20, 2020 **Abbreviations:** COVID-19 = coronavirus disease 2019; NYC = New York City. * ED visits for ILI reported by date of visit, confirmed cases by date of diagnosis, and sentinel specimens by date of collection.

The estimated SARS-CoV-2 prevalence among patients of all ages in the target population was 0.3% during the week of March 1 and 11.3% during the week of March 8, with an estimated 15 and 1,170 undetected SARS-CoV-2 infections among patients of all ages in the target population during each respective week (Table). The estimated SARS-CoV-2 prevalence among patients aged <18 years in the target population was 2.0% during the week of March 8 and 17.7% during the week of March 15, with an estimated 103 and 227 undetected SARS-CoV-2 infections among patients aged <18 years in the target population during each respective week (Table). During the weeks of March 1 and March 8, there were 26 and 1,917 confirmed cases of COVID-19, respectively, in NYC among persons of all ages. During the weeks of March 8 and March 15, there were 42 and 457 confirmed cases of COVID-19, respectively, in NYC among persons aged <18 years (Table).

Full genome sequences were generated from all 36 positive SARS-CoV-2 specimens. All sequences fell across three arbitrarily defined groups (A, B, and C) (Supplementary Figure, https://stacks.cdc.gov/view/cdc/90347). Two of the NYC sequences clustered in Group A, which contains sequences primarily derived from cases diagnosed in patients in the United States, who were mostly from the state of Washington, and includes other sequences from New York. Seven sequences clustered in Group B, which includes early sequences detected in China and other global sequences, as well as other sequences from New York. The remaining 27 sequences most closely clustered with New York sequences within Group C, which was largely dominated by sequences detected in Europe and North America.

## Discussion

During March 5–14, at approximately the same time as specimens from sentinel surveillance among persons with ILS in NYC were being collected, public health officials in Santa Clara County, California found SARS-CoV-2 prevalence to be 11% in specimens that tested negative for influenza collected from patients of all ages at four sentinel urgent care sites (*6*); in addition, 5.3% of patients with no known travel exposure or contact with a traveler, who were evaluated for mild influenza-like illness March 12–13 and March 15–16 at one medical center in Los Angeles, had positive test results for SARS-CoV-2 (*7*). Both Santa Clara County and Los Angeles used an identified surveillance approach that included collecting patient information on age, sex and travel history, whereas New York City used a deidentified approach. Differences in sampling methods and populations therefore limit direct comparisons; however, value can be found in recognizing various approaches to conducting sentinel surveillance.

During the weeks of March 8 and March 15, there was an increase in confirmed cases of COVID-19 among persons aged <18 years in NYC. During this same period, DOHMH estimated an increase in prevalence and undetected cases of COVID-19 among persons aged <18 years with ILS and negative influenza test results. These reported and estimated increases suggest that further investigation is warranted into the role children play in community transmission and the effect school closures might have as a mitigation strategy.

The sequence from March 2, 2020, (the earliest positive sentinel specimen collected) clustered with early sequences from Europe and United States (Group B), which also cluster with sequences from China. No sentinel sequences were directly connected to sequences from Wuhan, China, where the outbreak originated. This was unanticipated, given that most sentinel EDs were used by patients residing in ZIP codes with a high proportion of Chinese speakers. Rather, the sequence analysis suggests probable introductions of SARS-CoV-2 from Europe, from other U.S. locations, and local introductions from within New York. Domestic airport screening and bans on foreign nationals traveling from China were implemented on February 2;*** however, similar travel restrictions from the Schengen Area in Europe were only implemented March 13.^†††^ Although travel restrictions are an important mitigation strategy, by the time the European restrictions were implemented, importation and community transmission of SARS-CoV-2 had already occurred in NYC.

Based on target population calculations, many SARS-CoV-2 infections likely went undetected during the surveillance period in NYC. Expanding the testing criteria at the beginning of the outbreak to include persons with any travel exposure and with ILS without an alternative diagnosis would have increased the number of cases detected through passive surveillance. Limited testing capability and strict testing criteria led to many COVID-19 cases going undetected, slowed DOHMH’s capacity to use surveillance to make timely public health decisions, and ultimately contributed to sustained community transmission (*1*).

The findings in this report are subject to at least six limitations. First, the deidentified surveillance approach precluded collection of epidemiologic information, including any personal identifiers, demographic information, travel and exposure history, and specific sentinel ED, to support interpretation of the genetic links among specimens or further investigate clusters. Second, the change in age eligibility criteria during the surveillance period limited comparisons across weeks. Third, the pooling approach to laboratory testing has the potential to dilute low viral load samples leading to a false-negative result. Fourth, the small number of patients tested led to large uncertainty in estimated SARS-CoV-2 prevalence and the number of undetected COVID-19 cases in the target population. Fifth, a population survey to estimate the number of infected persons with ILS who did not seek medical attention was not completed until later in the pandemic, so these data could not be used to estimate infection prevalence among the general NYC population. Finally, the potential bias introduced by the sentinel sites selected and populations served affected the generalizability of these findings.

Sentinel surveillance and genetic sequencing, if available early after the emergence or reemergence of a new disease, can guide public health response strategies. DOHMH urges jurisdictions to leverage existing or new infrastructure to establish sentinel surveillance and specimen sequencing in preparation for a subsequent wave in the COVID-19 pandemic and for future outbreaks.

**Summary**

**What is already known about this topic?**

To limit SARS-CoV-2 introduction, the United States restricted travel from China on February 2 and from Europe on March 13, 2020. By March 15, community transmission was widespread in New York City (NYC).

**What is added by this report?**

The NYC Department of Health and Mental Hygiene conducted sentinel surveillance of influenza-like symptoms (ILS) and genetic sequencing to characterize community transmission and determine the geographic origin of SARS-CoV-2 infections. Among 544 specimens tested from persons with ILS and negative influenza test results, 36 (6.6%) were positive. Genetically sequenced positive specimens most closely resembled sequences circulating in Europe.

**What are the implications for public health practice?**

Partnering with health care facilities and establishing systems for sentinel surveillance with capacity for genetic sequencing before an outbreak can inform timely public health response strategies.

## Footnotes

* Influenza-like illness is defined as having fever and either cough or sore throat recorded in the ED chief complaint.

† https://www1.nyc.gov/assets/doh/downloads/pdf/hcp/weekly-surveillance03072020.pdf.

§ Influenza-like symptoms are defined as having at least one of the following signs or symptoms recorded in the ED chief complaint: chills, fever, upper respiratory infection, cough, sore throat, runny nose, congestion, headache, or fatigue.

¶ Specimens from all EDs were negative for influenza by polymerase chain reaction testing, two EDs required a negative respiratory syncytial virus test, and two additional EDs required a negative respiratory viral panel.

** Table S1601 (Language Spoken at Home), per American Community Survey, 2013–2017, available at https://data.census.gov/cedsci/.

§§ Target population size = ED visits for ILS citywide x (ED visits for ILS at sentinel sites with influenza tests performed/ED visits for ILS at sentinel sites) x (ED visits for ILS at sentinel sites with negative influenza test results/ED visits for ILS at sentinel sites with influenza tests performed).

¶¶ https://epitools.ausvet.com.au/trueprevalence.

*** https://www.whitehouse.gov/presidential-actions/proclamation-suspension-entry-immigrants-nonimmigrants-persons-pose-risk-transmitting-2019-novel-coronavirus/.

††† https://www.whitehouse.gov/presidential-actions/proclamation-suspension-entry-immigrants-nonimmigrants-certain-additional-persons-pose-risk-transmitting-2019-novel-coronavirus/.
